# A mixed‐methods pilot randomized control trial of ultrasound visual biofeedback versus standard intervention for children with cleft palate ± cleft lip: Parents’ and children's perspectives

**DOI:** 10.1111/1460-6984.13144

**Published:** 2024-12-09

**Authors:** Joanne Cleland, Robyn McCluskey, Marie Dokovova, Lisa Crampin, Linsay Campbell

**Affiliations:** ^1^ Department of Psychological Sciences and Health University of Strathclyde Glasgow UK; ^2^ Royal Hospital for Children NHS Greater Glasgow and Clyde Glasgow UK

**Keywords:** acceptability, articulation intervention, cleft palate ± cleft lip, ultrasound

## Abstract

**Background:**

Ultrasound visual biofeedback (UVBF) has the potential to be useful for the treatment of compensatory errors in speakers with cleft palate ± lip (CP±L), but there is little research on its effectiveness, or on how acceptable families find the technique. This study reports on parents’ and children's perspectives on taking part in a pilot randomized control trial of UVBF compared with articulation intervention.

**Aims:**

To determine the acceptability of randomization, UVBF and articulation intervention to families. We set feasibility criteria of at least 75% of responses rated as acceptable or positive in order to determine progression from a pilot to a full randomized control trial.

**Methods & Procedures:**

A total of 19 families who received UVBF therapy (11 families) and articulation intervention (eight families) were invited to participate. Mixed methods were employed: two questionnaires to determine the acceptability of UVBF and articulation intervention, respectively; and semi‐structured focus groups/interviews. Questionnaires were analysed for frequency of positive versus negative acceptability and the focus groups/interviews were analysed using thematic analysis and coded using the theoretical framework of acceptability.

**Outcomes & Results:**

More than 75% of families rated randomization as acceptable and more than 75% of families rated both interventions as acceptable, with the caveat that half of the participants did not wish to continue articulation intervention after the study. For some families, this was because they felt further intervention was not required. Six families (three in each intervention) volunteered to take part in the focus groups/interviews. Results showed more positive than negative themes regarding acceptability, particularly affective attitude where high levels of enjoyment were expressed, although some participants found the articulation intervention ‘boring’. In both groups, there was a considerable burden involved in travelling to the hospital location.

**Conclusions & Implications:**

Randomization in a clinical trial is acceptable to families; UVBF and articulation intervention are acceptable and indeed enjoyable. The burden of the additional outcome measures required for a clinical trial is manageable, although there is a travel burden for participants. Future studies should seek to mitigate the travel burden by considering additional locations for intervention.

**WHAT THIS PAPER ADDS:**

**What is already known on the subject**

- UVBF shows potential for teaching children with speech sound disorders new articulations. Previous studies looking at the acceptability of using ultrasound in speech intervention have only asked for the drawbacks of the intervention: finding the ultrasound gel to be cold and sticky and the probe to be uncomfortable. SLTs, however, believe that the technique offers advantages due to its ability to visualize tongue movements.

**What this paper adds to the existing knowledge**

- This study asks parents and children for their full views about using ultrasound in speech intervention and compared this to articulation intervention. We also asked parents how they feel about being randomized to one of these interventions. Results were positive for both interventions, with parents highlighting the importance of intelligible speech to their child. Children enjoyed both interventions, though the articulation intervention could be ‘boring’ or ‘repetitive’. For all families, there was a considerable burden travelling to clinics.

**What are the potential or actual clinical implications of this work?**

- Clinicians can be assured that both articulation therapy and ultrasound therapy are acceptable to families. Efforts could be made to ensure that articulation intervention is not repetitive and that appointments are offered at locations which are accessible to families where possible.

## INTRODUCTION

Cleft palate ± lip (CP±L) is a common congenital craniofacial difference, with an incidence of one in 700 births in the UK (Bellis & Wohlgemuth, 1999). The key treatment is surgical; however, problems producing intelligible speech often occur and in some children cleft speech characteristics persist, requiring intervention from a speech and language therapist (SLT) (Medina et al., 2019). This speech difficulty can lead to social and educational consequences, with the speech of children with CP±L rated as more likely to belong to someone who is ‘unhealthy’, has ‘no friends’ and is ‘ugly’ (Lee et al., 2017).

Intervention options for children with CP±L who have compensatory (active) errors usually fall into two broad categories: articulatory (also known as motor‐phonetic) approaches and phonological approaches (Bessell et al., 2013). In articulatory approaches, compensatory errors are considered as mis‐learnings of a motor programme for a specific speech sound which arises due to an effort to circumvent anatomical differences. For example, anterior plosives are backed to compensate for a difficulty achieving adequate oral pressure. Anterior plosives such as /t/ may therefore be realized as [k] or indeed as non‐oral speech sounds such as glottal stops. In the former example, a homophony then occurs between words contrasting /t/ and /k/ (e.g., ‘key’ and ‘tea’ are both pronounced as [ki]). This contrast collapse also lends itself to a phonological intervention approach where the child is taught the phonological rules and patterns surrounding such contrasts. A recent study by Alighieri, Bettens et al. (2020) compared these interventions in a randomized design and found that both interventions led to improvements in children with CP±L, but that the phonological approach had an advantage in terms of consonant proficiency.

Nevertheless, several recent studies suggest that articulatory approaches can be effective, especially when they employ the principles of motor learning (Hanley et al., 2023). Moreover, the distinction between articulatory and phonological approaches is likely a false dichotomy since children must both learn a new motor programme for a sound in error and then generalize this to other linguistic units. Generalizing a new motor program can be extremely challenging for children with articulation disorders when they are not stimulable for the speech sound in question. For children with CP±L, achieving anterior articulations is often a difficulty. The motor learning literature describes this part of learning any new motor skill as ‘acquisition’ (Maas et al., 2008), after which the learner must practice the new speech sound in order to retain it and then finally generalize its use. During the acquisition phase, knowledge of performance is particularly useful. This is provided by giving the learner specific information about the articulatory movements, for example, ‘good, I saw your tongue moving to the front of your mouth’. This is challenging for speech because the main articulator, the tongue, is largely hidden from view. However, instrumental articulatory techniques can be used to gain direct access to the movement of the articulators, thus giving more accurate knowledge of performance to both the SLT and the child with CP±L. In the CP±L literature there is a long history of using electropalatography (EPG) as a biofeedback tool to provide children with CP+/L knowledge of performance regarding articulatory placement (Lee et al., 2009). More recent studies using EPG also incorporate phonological principles (Patrick et al., 2023). However, EPG is expensive and logistically difficult because each speaker requires a custom‐made pseudo‐palate. An alternative articulatory technique, ultrasound visual biofeedback (UVBF) has been used successfully with children with a variety of other types of speech sound disorders (Sugden et al., 2019) and is predicted to become an important technique for the treatment of cleft speech characteristics (Cleland, 2023).

### UVBF

In this technique, an ultrasound probe is placed under the chin of the speaker and used to image the tongue in either a mid‐sagittal or coronal view (Cleland, 2021). For intervention the technique is used most often in the mid‐sagittal view and is particularly useful in the acquisition stage of intervention, that is, for teaching new articulations. In the case of speakers with CP±L, this is most likely to be anterior articulations. Only a small number of studies have used ultrasound with speakers with CP±L (see Cleland, 2023 for an overview) and most of these studies have not used it for intervention. For example, Bressmann et al. (2011) used ultrasound to illustrate a covert error where attempts at /k/ were perceived as glottal stops but were actually simultaneous pharyngeal plus glottal stops. Identification of these types of errors is important therapeutically because the SLT can give more precise information to the speaker about what is required to remediate the error. In another study, Cleland et al. (2020) used ultrasound to classify speech errors and observed covert errors such as double articulations and retroflexion in 39 children with CP±L. They report that inter‐transcriber reliability was higher when transcribers used ultrasound in addition to traditional auditory methods, suggesting it could be a useful objective assessment method.

Only a small number of studies have used UVBF for intervention with speakers with CP±L. Three intervention studies, all case studies with five or fewer participants, trialled ultrasound as a biofeedback tool in children with CP±L and showed improvements in accuracy of targeted consonants (Hashemi Hosseinabad & Xing, 2024; Parks, 2018; Roxburgh et al., 2021). None of these studies report on how acceptable the children and their carers found this intervention. Acceptability of an intervention can be operationally defined as a ‘multi‐faceted construct that reflects the extent to which people receiving or delivering a healthcare intervention consider it to be appropriate based on anticipated or experienced cognitive and emotional responses to the intervention’ (Sekhon et al., 2017). Acceptability is important because patients are more likely to adhere to interventions which are acceptable. Moreover, UVBF is a novel intervention which involves the use of instrumentation that children are unfamiliar with, thus we cannot assume it is acceptable to children and their carers. The current study reports on the participant and carers’ views on UVBF intervention compared with articulatory treatment as part of a pilot feasibility randomized control trial comparing these two interventions (Cleland et al., 2022). Quantitative results of the pilot will be reported separately. Seeking patients’ and carers’ views has largely been neglected in speech intervention studies and specifically in studies using instrumentation. Notable exceptions to this are several recent studies by Alighieri and colleagues (Alighieri et al., 2021, 2023; Alighieri, Peersman et al., 2020) and Sell et al. (2023). All these studies suggest that parents judge speech intelligibility to be very important to their children and they expect this to be the goal of intervention; however travelling to appointments and the opportunity cost of intervention can be high and high‐intensity intervention can lack variation (Alighieri et al., 2023). Sell et al. (2023) also suggest that parent‐led intervention is acceptable to parents, which is important as many interventions, whether parent or clinician led, involve home practice. None of these studies looked at the acceptability of an instrumental method such as ultrasound, which although medically non‐invasive, could be considered by participants to be more invasive than non‐instrumental techniques.

There are also few studies that have investigated the experiences of patients and SLTs with UVBF. In 62 children without CP±L, Preston et al. (2018) used a simple two‐part questionnaire to ask about the negative aspects of UVBF and children reported minor inconveniences such as the ultrasound gel being cold or sticky and the ultrasound probe being annoying. They did not ask the participants what the positive aspects (if any) of the intervention were or use any theoretical framework to look at the acceptability of the technique. In a separate study, Dugan et al. (2023) interviewed seven SLTs with experience of UVBF about the barriers and benefits of using the technique. The top identified barrier was a lack of access to the equipment and the most frequently mentioned benefit was the ability to visualize articulatory responses to cues live on screen. There is therefore a gap in understanding parent and child's perspectives towards using UVBF, particularly whether there are any positive perspectives. This study seeks to address this gap, while also understanding how the acceptability of UVBF compares to treatment as usual, which in this case was articulation intervention.

Given this is a pilot feasibility study, we asked participants about both interventions and we asked about the acceptability of randomization and the additional assessment sessions required for a clinical trial (pre‐, post‐ and follow‐up). Results will be used to inform a future larger trial and we do not therefore seek to present a definitive comparison of UVBF and articulation therapy. A mixed methods design incorporating questionnaires for all participants and opt‐in focus groups for parents and children over 12 was used to understand the acceptability of UVBF compared with standard treatment.

The overall study has a number of feasibility objectives and criteria which will be used to determine whether progression to a full trial is warranted (Cleland et al., 2022). We set a feasibility threshold of at least 75% in line with a similar study by Pennington et al. (2020). Those relevant to the current report of parents’ and children's perspectives were:

- To determine the acceptability of randomization to children and their families.
  ▪ 75% of children and their families rate randomization as acceptable in a questionnaire.
- To determine the acceptability of UVBF as an assessment tool and intervention tool.
  ▪ 75% of children and their families rate ultrasound as an acceptable technique in a questionnaire.
  ▪ Focus group analysis contains more positive than negative themes regarding acceptability (of ultrasound—note we also report the acceptability of articulation intervention in this report).

## METHOD

This study was approved by the West Midlands–South Birmingham Research Ethics Committee (21/WM/0104). The trial was registered on the 22 March 2021 ISRCTN17441953 and the protocol was published before data collection (Cleland et al., 2022).

### Participants

Children aged 4;6–16 with a syndromal or non‐syndromal palatal cleft of any type were invited to participate in the study. We set the minimum age as 4;6 because prior research with UVBF begins around school age (Sugden et al., 2019), which is 4;6 in Scotland. Children were eligible if they had at least one speech error that would normally be a candidate for articulation intervention, for example, backing. Children with a bilateral hearing loss of greater than 30 dB, planned surgery within the next 3 months, or severe language delay were excluded. Following baseline, the children were randomized in a 1:1 ratio, stratified for age into either the UVBF or articulation arm of the trial. Overall, we recruited 19 children for the trial. See Table 1 for participant details. All families were invited to take part in online focus groups about their experiences of the intervention and taking part in the trial.

**TABLE 1:** Participant details.

**Participant ID**	**Treatment**	**Age (years; months)**	**Sex**	**Additional diagnoses**	**Language spoken at home**	**Other languages spoken**	**Years in Scotland**	**Accents spoken at home**	**Type of cleft**
SS_01_F_07	Ultrasound	7;4	F	n.a.	English	n.a.	7	North‐east Scottish and Glaswegian	UCLP
SS_02_M_12	Ultrasound	12;11	M	n.a.	Portuguese, English	French (not fluent)	12	Portuguese	BCLP
SS_03_M_07	Articulation	7;3	M	n.a.	English	n.a.	7	Scottish	CP
SS_04_M_05	Ultrasound	5;5	M	n.a.	English	n.a.	5	Scottish	UCLP
SS_05_M_05	Articulation	5;7	M	n.a.	English	n.a.	5	Scottish	UCLP
SS_06_F_09	Ultrasound	9;10	F	n.a.	English, Filipino	Not reported	Not reported	Not reported	UCLP
SS_07_M_08	Ultrasound	8;9	M	n.a.	English	n.a.	8	Scottish	UCLP
SS_08_M_07	Ultrasound	7;6	M	Asthma	English	n.a.	3	Scottish	submucous cleft
SS_09_M_15	Ultrasound	15;6	M	n.a.	English	n.a.	15	Scottish	BCLP
SS_10_F_05	Articulation	5;2	F	n.a.	English	n.a.	5	Scottish	CP
SS_11_M_07	Ultrasound	7;6	M	n.a.	English	n.a.	6	Scottish	BCLP
SS_12_F_05	Ultrasound	5;0	F	n.a.	English	n.a.	5	Scottish	BCLP
SS_13_F_05	Articulation	5;8	F	Stickler Syndrome, hearing loss	English	n.a.	5	Mum Scottish, dad Geordie	CP
SS_14_F_04	Articulation	4;6	F	Stickler Syndrome, hearing loss	English	n.a.	4	Mum Scottish, dad Geordie	CP
SS_15_M_07	Articulation	7;11	M	Hearing impairment	English	n.a.	7	Scottish	UCLP
SS_16_M_06	Ultrasound	6;5	M	n.a.	English	n.a.	6	Scottish	CP
SS_17_F_10	Articulation	10;4	F	n.a.	English	n.a.	10	Scottish	UCLP
SS_18_M_11	Articulation	11;9	M	22q11 deletion syndrome	English	n.a.	11	Mum/brother Scottish, dad Bristolian	CP
SS_19_M_11	Ultrasound	11;8	M	n.a.	English	n.a.	11	Mum Scottish, dad English	BCLP

### Interventions

Details of the interventions are reported in the protocol (Cleland et al., 2022). In short, we aimed to make the interventions as similar to each other as possible in terms of dosage and agent of intervention. Articulation intervention mostly involves modelling and imitation strategies from the SLT and children are encouraged to listen to their own speech and make corrections. Games may be incorporated to ensure high levels of repetition of targets. UVBF involves wearing an ultrasound probe stabilizing headset and looking at live tongue movements on a computer screen (a video of an ultrasound therapy session can be seen here https://speechstar.ac.uk/ultrasound‐therapy‐videos/). The SLT uses this information to help the child correct their speech movements. Both groups received six sessions of intervention, one per week for 6 weeks, lasting around 45 min at the city centre hospital they normally attend with the SLTs who are normally responsible for their care. Home practice was encouraged. One pre‐intervention assessment (1 week before intervention) and two post‐intervention assessments (2 and 3 months post‐randomization) were conducted at a city centre university by a research SLT employed on the project (the third author). Pre‐ and post‐assessments were optionally conducted over Zoom as requested.

### Data collection and analysis: Questionnaires

Three months after randomization, at the last follow‐up assessment session parents/carers were invited by the research SLT to complete a questionnaire designed to determine whether taking part in the intervention study was acceptable to families. Different questionnaires were distributed via the online programme Qualtrics to both intervention groups. The questionnaires are available via https://pureportal.strath.ac.uk/en/datasets/sonospeech‐data‐collection‐instruments/. Questions 1–3 asked about the acceptability of randomization and the additional outcome assessments. We coded these as positive, negative or neutral (e.g., ‘Your child was randomly allocated to receive articulation therapy. Were you happy with this allocation? Yes = Positive, No = Negative, Not sure = Neutral’). Questions 4 and 7 (a. and b. for the ultrasound group) and 8 asked about the enjoyment of the intervention and question 9 asked about the continuation of the intervention. Percentages of positive and negative responses are presented as overall percentages and compared with the feasibility thresholds of at least 75% of positive responses. Questions 5, 6 and 9 were free text comments about likes/dislikes of the interventions and reasons for continuation/termination. These were subjected to a content analysis with deductive coding in line with the theoretical framework of acceptability (Sekhon et al., 2017) using a similar method to Alighieri et al. (2023). This framework comprises seven constructs: affective attitude, burden, perceived effectiveness, ethicality, intervention coherence, opportunity costs and self‐efficacy. Each of these assesses how an individual perceives an intervention's appropriateness and acceptability. For example, the construct ‘affective attitude’, is defined as ‘how participants feel about the intervention’, for example, whether it was enjoyable. Likewise, ‘burden’ is how much effort is involved in taking part in the intervention and might be affected by practicalities such as number of intervention sessions, location of session and so on. Definitions for each construct are given in Table 4. The paper describing the development of the theoretical framework of acceptability (Sekhon et al., 2017) suggests that it could be used in deductive analysis of focus groups or interviews. Here we use it for both focus groups (see below) and for the free text of our questionnaires. Although focus groups (below) yield richer data than free text comments in questionnaires, we present both since all participants in the study completed the questionnaire, but only some participants took part in the focus groups. We coded statements such as ‘definitely enjoys the sessions’, or ‘I thought it was excellent’ as positive feelings. The first author performed the deductive coding and this was checked by the other authors.

Both groups were also asked to complete the Experience of Service questionnaire (Brown et al., 2014), which is a standard measure used in the clinical service to measure patient satisfaction with the service generally. This questionnaire comprises two composite scores: satisfaction with care and satisfaction with the environment. We report group means for each of these.

### Data collection and analysis: Focus groups

Parents/carers and children over 12 were invited to join online focus groups at the completion of the project to discuss their experiences. These were presented to families as an additional, optional part of the research and were organized at times convenient to participants. Focus groups were conducted separately for each arm of the trial. Due to scheduling conflicts, the group for the articulation arm of the study had to be conducted as individual semi‐structured interviews. Three families including one child in the articulation group agreed to join and three families in the ultrasound group agreed. The child was present for the duration of the focus group and specific questions were directed at them towards the end of the session. Focus groups/interviews were conducted online by a qualitative researcher (the second author) who was not otherwise involved in the study. This researcher is a clinical psychologist with no prior knowledge of speech and language intervention and CP±L. A member of the research team (the first author) observed the focus groups/interviews but did not participate. The topic guide for both focus groups/interviews is available via https://pureportal.strath.ac.uk/en/datasets/sonospeech‐data‐collection‐instruments/. The groups/interviews were semi‐structured in nature with open‐ended questions. The focus groups/interviews were recorded in Microsoft TEAMS, transcribed verbatim and identifying information removed. The analysis comprised two stages, first inductive thematic analysis and then deductive coding using the theoretical framework of acceptability. We report the results of both. The clinical psychologist qualitative researcher (the second author) performed the inductive thematic analysis, following the same methods employed in a similar study with children with cerebral palsy (Pennington et al., 2020). Once the inductive analysis was complete, the themes arising from it were then deductively coded by the first author into the seven constructs of the theoretical framework of acceptability (Sekhon et al., 2017), tabulated and checked by all authors.

### Reflexivity and trustworthiness

Several strategies were employed to enhance the trustworthiness of the findings. First, a researcher who was not familiar with the overall study and had no experience with people with CP±L conducted the focus groups/interviews and performed the inductive analysis. This allowed us to minimize pre‐conceptions about the effectiveness of the either intervention or the design of the study. All the authors critically examined the interpretations and conclusions drawn from the data. The first author is a researcher with a specific interest in ultrasound biofeedback, she reflected on this potential bias throughout the process, particularly when comparing the interventions. No changes were made to the thematic analysis by the first author or any of the other authors. To enhance transferability, we include a detailed description of the research setting, participants and data‐collection processes, which were in line with our published protocol. We maintained an audit trail throughout the research process. No changes were made to the original protocol, with the exception of the addition of deductive coding into the Theoretical Framework of Acceptability (Sekhon et al., 2017), which we consider a useful way of summarizing findings.

## RESULTS

Results are presented first for the questionnaire data, grouped by intervention: articulation intervention, then ultrasound intervention. We then present the deductive thematic analysis of the focus groups/interviews, again by intervention type. Lastly, we summarize the free text comments from the questionnaires and the thematic analysis of the focus groups/interviews for both types of intervention by applying deductive coding into the Theoretical Framework of Acceptability and presenting summary results in a table.

### Questionnaire: Articulation intervention

A total of 15 families^1^ (83% response rate) responded to the post‐intervention questionnaire. Of these nine were in the ultrasound group and seven in the articulation group. The parents of one child in the articulation group also had a child in the ultrasound group. They completed only one Qualtrics questionnaire and said their answers would be no different for the child in the ultrasound group. All responded to the Experience of Service questionnaire. Table 2 presents the results of the questionnaire for the articulation therapy group. Results met the threshold of at least 75% positive responses, except for the continuation of intervention. For the free text questions (Q5, Q6, Q9), most participants (four) responded that they enjoyed playing games, two that they enjoyed the different techniques/exercises, two liked the interaction with the SLTs and one liked the improvements in their child's speech. Question 6 asked what they did not like about articulation therapy. Most (five participants) said ‘nothing’, one said that the intervention was challenging and one disliked the burden of travel. Finally, question 9 asked participants if they wished to continue articulation intervention. Most (four) responded yes because further improvement was required. Two responded no, as the children had already improved and a further two had other priorities at that time. Note, one participant responded that they had other priorities, but would like to undertake additional intervention in the future. A summary of these responses coded into the theoretical framework of acceptability is presented and discussed alongside the results for the focus groups in Table 4.

**TABLE 2:** Articulation intervention questionnaire results.

	**% Responses**	
**Articulation intervention group**	**Positive**	**Negative**
Acceptability of randomization and additional assessments (Q1, 2, 3)	95%	5%
Enjoyment of articulation intervention (Q4, Q7, Q8)	86%	14%
Continuation of articulation intervention (Q9)	57%	43%

### Questionnaire: Ultrasound intervention

Table 3 presents the results of the questionnaire for the ultrasound therapy group. This group also met the threshold of at least 75% positive responses. Results are similar to the articulation group, with the exception of the continuation of the intervention, which was more positive in this group. In terms of free text comments, most participants (seven families) responded that they enjoyed seeing the tongue movements and the visualization on the screen (six participants). One participant commented that they liked seeing the improvements because they ‘learned more’. In terms of dislikes, for most participants, this was ‘nothing’ (six participants), but two participants disliked the gel and three disliked the headset, although all with the caveat that the headset was not worn for long periods, suggesting it was tolerable. Finally, question 9 asked participants if they wished to continue ultrasound intervention. All responded that they would, with nine suggesting this was because further intervention was required and two responding that they thought this intervention was better than other interventions.

**TABLE 3:** Ultrasound intervention questionnaire results.

	**% Responses**		
**Ultrasound intervention group**	**Positive**	**Negative**	**Neutral**
Acceptability of randomization and additional assessments (Q1, 2, 3)	94%	0%	6%
Enjoyment of ultrasound intervention (Q4, Q7a, Q7b, q8)	86%	11%	9%
Continuation of ultrasound intervention (Q9)	100%	0%	0%

### Questionnaire: Experience of service

In both groups, the ‘experience of care’ scores met the ceiling of 100% satisfaction. This part of the questionnaire relates to the interpersonal interaction with the treating clinicians, for example, ‘I was treated well by the people who have seen my child’. In the articulation group, the score for satisfaction with the environment was 94% (SD = 8%) and in the ultrasound group it was 91% (SD = 11%). These slightly lower scores reflect minor dissatisfaction with either travelling to the hospital or the timing of appointments.

### Interviews: Articulation intervention, inductive analysis

Data was coded and extracted using thematic analysis. Four themes emerged from the data: (1) accessibility to therapy regarding transport, social and everyday difficulties; (2) enjoyment of articulation therapy; (3) effectiveness of articulation therapy on speech; and (4) efforts of practising outwith therapy. Examples are presented for each theme below:

### Theme 1: Accessibility to therapy regarding transport, social and everyday difficulties

Participants recognized the struggles in participating in the therapy, in particular, the distance needed to travel to appointments:

It was a bit of a pain. It didn't bother me, but the wee one was fed up. (Articulation group parent 1)

Following this, the frustration of the child during travel is shown:

It was you just listening to him moaning about it. (Articulation group parent 1)

Parent 2 used words such as ‘pain’, ‘fed up’ and ‘moaning’ to describe the travel for the child even though the child themselves stated the travel did not bother them showing the contrast in feelings. Distance factored into whether some participants completed the pre‐ and post‐assessment measures online or in person:

It's just time‐wise trying to get from work to the university. There just wasn't enough time in the day to. (Articulation group parent 2)

This point was exasperated by external factors such as childcare:

When it gets to that point, it just becomes a bit harder and you're I suppose intruding a bit, do you know what I mean, when you've got to start looking for help (Articulation group parent 1)

Parent 1 discussed the point of looking for help from others, stating this also makes it harder for participation, additionally when asked if a single parent may find participating more challenging parent 2 stated ‘Yes, definitely they would struggle’ (Articulation group parent 2). With barriers affecting those with less support, participants suggested ways to recruit future participants such as being ‘flexible’ with times and days and particularly in shortening the distance to travel.

If people are quite far away and could maybe struggle to get to you, it would be better if you could maybe meet even if you could meet halfway somewhere (Articulation group parent 2)

Schools were suggested as an effective meeting point as ‘schools would be onboard cause they're the same they want to help’ (Articulation group parent 3), showing the positive relationship that the parents have with this environment in comparison to a hospital where ‘kids do look on these places, they don't go there for a good reason’ (Articulation group parent 1).

Another issue brought up within the interview showed the range of difficulties in participation, which regarded the online and technological aspects of the study. Participants showed their struggle in filling out online forms (including the questionnaires) with a lack of knowledge on being tech involved.

I'm not very computer‐friendly so I found it quite difficult trying to do the forms online (Articulation group parent 2).

Parent 2 struggled to complete the forms. When using a postal letter with a return envelope was suggested the participant stated, ‘That would be a bit easier for me’ (Articulation group parent 2) showing a different form of contact was more effective for the participant.

### Theme 2: Enjoyment of articulation therapy

Despite the travel issues, the overall outlook from parents and carers was positive:

I think it worked well … they tried to implement an element of fun to it, which is great because otherwise they're not really going to get the wee ones to engage the same way. (Articulation group parent 1)

Parents and carers expressed how the therapy worked well, also elements of ‘fun’ were implemented for engagement keeping parents and carers ‘quite happy with it’ (Articulation group parent 2). Due to the therapy being the standard care for this group, there were also comments on the therapy being ‘just what we were used to’ (Articulation group parent 2). Though parents and carers overall stated the study as ‘perfect’ (Articulation group parent 3) and ‘it has certainly been worthwhile’ (Articulation group parent 1).

The child's perspective showed mixed feelings towards the study and therapy. The child interviewed stated the therapy as ‘it's boring’ (Articulation group child 1) and when asked what would make the therapy better the solution was ‘don't do it’ (Articulation group child 1). Although the child found the therapy itself boring when asked if he liked the SLTs the child answered positively. Furthermore, when asked about the games and if he enjoyed these he answered ‘sometimes’ (Articulation group child 1). Parents and carers emphasized how the children felt about the therapy:

He was excited to go. Em he wasn't keen on the stickers.^2^ (He didn't). He was above that. Do you know he was coming 7, he was getting above that. (Articulation group parent 3)

With the therapy becoming almost routine for the children the parents incentivised attendance: ‘he kind of took it as a little day trip, really, to go and get his cake and his drink. Go and see them play some games’ (Articulation group parent 2). However, parents and carers stated that the child found the therapy ‘repetitive and a bit kind of boring’ (Articulation group parent 1) also the format in which the therapy was conducted showed importance, ‘he much preferred face to face’ (Articulation group parent 2) as ‘he doesn't like sitting on the screen’ (Articulation group parent 2). The children showed mixed responses to the therapy overall from positive to repetitive.

### Theme 3: Effectiveness of Articulation therapy on speech

Parents believed the intervention was effective:

I'm glad that D got the opportunity, and his speech is phenomenal now you know, there's nothing no barriers for him. (Articulation group parent 3)

Also stating ‘Well, you can't get any better than speaking better’ (Articulation group parent 3) shows the extent of impact on the child's speech. The therapy also impacted the confidence of the child ‘he says, you know, I can say my name and say my whole name and people know who what my name is. So, for him, that was a huge thing’ (Articulation group parent 3). Though the therapy helped some children there were some who did not see a difference in speech stating:

My mum (the child's grandmother) sometimes struggles, and she'll sort of, say, look at me, like for me to interpret basically. So I think possibly. I mean, everybody every individual's different, but for us we probably would have needed more (Articulation group parent 1)

Outside factors are also evident in the success of the therapy:

I don't know whether it was just because he had the surgery first and then the speech therapy then helped increase what happened from the surgery, but it has definitely helped over the years. (Articulation group parent 2).

Parent 2 demonstrates a clear variable as the child experienced a cleft‐related surgery before the therapy began. This created confusion as to the causation of improved speech.

### Theme 4: Efforts of practising outwith therapy

The participants expressed how they practised as well as the struggle to engage in practice. They used any moment they could to practice ‘We didn't spend loads of time on it at the one time, so it would be literally maybe a few minutes while I'm making dinner’ (Articulation group parent 1). Parent 1 shows difficulty in practising the sounds, stopping ‘the minute he started to disengage’. Also, due to the constant nature of practice and correcting speech participants sometimes resulted in saying ‘I don't know what you're saying. Try another word’. (Articulation group parent 2). Showing the frustration practice can cause.

However, with more practice outwith the therapy, there was a noticeable difference in speech:

We did it on the way up and the way down and D would just come through and practise too. Just randomly say things because the more he did it, the more he could see his speech was getting better (Articulation group parent 3)

Parent 3 discussed how the child could see the change in his speech, this was a big motivator for participating in the homework. Also, parent 3 discusses practising on the way up and down from therapy with less distractions.

### Focus group: Ultrasound intervention, inductive analysis

Three main themes emerged from this group: (1) transport and external issues in participation; (2) thoughts towards ultrasound therapy and added understanding of speech; and (3) effects of ultrasound therapy on speech.

### Theme 1: Transport and external issues in participation

Travel was an issue of participation as parent 4 stated, ‘It was like a four‐hour journey just to get there and back’ (Ultrasound group parent 4). To make the sessions worthwhile for those travelling a suggestion of ‘The appointments need to be a bit longer’ (Ultrasound group parent 4), more time in sessions and more sessions in general were suggested by participants. Participants suggested the addition of clinics or areas which can be used for sessions, ‘Obviously just more choice of locations’ (Ultrasound group parent 5).

Though travel was highly discussed another issue of participating was household dynamics and makeup:

it wasn't so bad that I've managed to get their dad to look after them when I was taking G, and M was at Nursery. But if there is days that they were sick, I was kinda stuck trying to work out how I was going to do both (Ultrasound group parent 4)

Parent 4 (single parent) discusses managing to have the children's dad look after siblings while she attended sessions. This and the following comment of being ‘stuck’ in doing both suggest the struggle created in getting to and from appointments with outside pressures.

### Theme 2: Thoughts towards ultrasound therapy and added understanding of speech

Even though there were difficulties in travel the response towards the therapy and study were overwhelmingly positive. Participants expressed their own feelings as well as their child's, respectively, with high recommendations:

it's been great, I couldn't be more positive about it or recommend it, you know, highly enough for those that are interested if I'm being honest (Ultrasound group parent 6)

When asked if there were any disadvantages of the therapy participants answered, ‘not that I can think of’ (Ultrasound group parent 5). The enjoyment of the children was also recorded:

He loved it and he loves actually he loves going. He likes seeing (the SLTs) and he likes chatting to people (Ultrasound group parent 6)

G loved it. G was more excited about getting to pick what he could click on the screen for the wrong answer and the right answer, but he was dead excited to come through every week (Ultrasound group parent 4)

Travelling to the university added to the enjoyment of the children: ‘F quite enjoyed the whole ‘she was going into a university’ and not a hospital. I think she quite enjoyed that’ (Ultrasound group parent 5).

From the enjoyment of the study came a further understanding of the therapy as a whole and added the element of visualization. M Ultrasound says, ‘the study was amazing. Like it was amazing, and it has helped me understand more’ (Ultrasound group parent 4) this understanding extended to the children taking part.

sometimes he did struggle just doing the speech therapy. Sometimes he did struggle to get the sounds, cause he didn't really know where his tongue should be…it just gives you that kind of visual and the understanding and you can see how hard (you know well) I could see how hard he was trying (Ultrasound group parent 6)

Parent 6 shows their child initially struggled with where to place their tongue. Whereas understanding improved when adding a visual element: ‘I think it's just because it's visual as well, you know that helps’ (Ultrasound group parent 5).

Continuing the visualization factor, participants suggested the use of a computer in the therapy helped in the response from the children and was a great aid in their speech development.

F was told that she was doing it wrong, she just couldn't get out of the loop or to change it for what she was getting asked to do, but the fact that somehow a computer she responds better to that is telling her no You need to do it, you know It seemed to help her (Ultrasound group parent 5)

Totally agree, totally agree. Like the way that he's had speech therapy in the past he's not kinda understood, whereas showing on a computer that where his tongue should be (Ultrasound group parent 4)

Participants agreed on the positive benefit of using a computer for understanding and development of speech sounds.

### Theme 3: Effects of ultrasound therapy on speech

Participants’ views on the effectiveness of the therapy on speech were mixed and to an extent contradicting. Participants first expressed improvement in speech:

F is a bit older but her speech has improved You know in the last 6/7 months (Ultrasound group parent 5)

you could, when it started, see just little improvements each time (Ultrasound group parent 6)

Though improvement noted was stated as being ‘little’, there was comment of notable difference during and after ultrasound therapy. Participants were asked whether they felt their child's speech had improved in using the ultrasound therapy, parent 6 stated, ‘The only improvement is he could understand or visualise it’ implying an improvement in her speech output was not measurable.

if I tell G Remember where your tongue goes, then yeah, you can understand what he's saying. But if I was not to remind him I don't think people would understand what he's saying (Ultrasound group parent 4)

I don't know if it's a combination of just getting older, seeing where her tongue was and really focusing because she does like to talk (Ultrasound group parent 5)

Parent 4 suggests that G is understandable when reminded about his tongue though is still not understood by many when the reminder is not put in place. Parent 5 adds to this stating that the progression of speech could be from multiple factors including seeing where her tongue was.

*Comparing interventions using the theoretical framework of acceptability: deductive analysis of questionnaire free text and all focus groups/interviews*.

In order to determine the overall acceptability of both interventions and of the overall study we performed a content analysis with deductive coding in line with the theoretical framework of acceptability (Sekhon et al., 2017) and with a similar method to Alighieri et al. (2023) who also used this framework. This framework was used deductively and was applied to both the free text comments in the questionnaires and the interviews/focus groups and took place once the inductive thematic analysis detailed above was complete. Results for both groups are presented in Table 4, with positive and negative comments indicated in each of the constructs. This analysis revealed that there were more positive than negative themes regarding acceptability for both types of intervention, particularly participants’ affective attitude; however, there were also some negative constructs, particularly the burden of travel.

**TABLE 4:** Results summary coded into the theoretical framework of acceptability (TFA).

**TFA construct**	**TFA definition**	**Articulation intervention**	**Ultrasound intervention**
Ethicality	The extent to which the intervention has a good fit with an individual's value system	+F Was worthwhile +F ‘Can't get better than speaking better’	+F Would recommend to others +F Study was ‘perfect’
Affective attitude	How an individual feels about the intervention, after taking part	+Q Enjoyed Games +Q Enjoyed interaction with SLTs +F Fun elements −F Found the online questionnaires difficult to access −F The intervention was boring and repetitive	+Q Enjoyed seeing tongue movements +F Enjoyment and excitement +F Enjoyed interaction with SLTs +F Enjoyment of visiting university
Burden	The amount of effort that was required to participate in the intervention	−Q Burden of travel −F Burden of travel −F Childcare burden for siblings −F Burden of homework/practice	−F Burden of travel −F Childcare burden for siblings
Opportunity costs	The benefits, profits or values that were given up to engage in the intervention	−Q Different priorities (education)	
Perceived effectiveness	The extent to which the intervention is perceived to have achieved its intended purpose	+Q No further intervention required +F Speech and confidence improved −F Improvement minimal, more intervention required.	+F Improved understanding of the child how to produce specific sounds compared with other interventions +F Improvements in speech
Self‐efficacy	The participant's confidence that they can perform the behaviour(s) required to participate in the intervention	+F Able to practice while travelling to therapy +F Practice is important	+F Increased understanding of tongue movements from parents
Intervention coherence	The extent to which the participant understands the intervention and how it works	+Q Different techniques and speech exercises	+F Children and parents can understand tongue movements

*Note*: +Q indicates a positive reflection of the theoretical framework of acceptability statement from the free text in the questionnaires; −Q indicates a negative reflection of the theoretical framework of acceptability statement from the free text in the questionnaires; +F indicates a positive reflection in the focus groups; and −F indicates a negative reflection in the focus group.

## DISCUSSION

This study sought to determine the acceptability of both a randomized study design and a novel intervention, UVBF, to children with CP±L and their families. While researchers have traditionally been concerned with the effectiveness of an intervention, the acceptability of interventions to families is key to both adherence to the intervention and to the feasibility of conducting large clinical trials of an intervention. We consider it especially important to consider the acceptability of novel instrumental interventions such as UVBF which are uncommon in speech and language therapy. In the case of ultrasound, this is particularly important as to our knowledge the only published study on parent perspectives of using ultrasound with child participants asked only about the undesired effects, finding that the ultrasound gel was ‘cold or sticky’ and the probe could be ‘uncomfortable or annoying’ (Preston et al., 2018). It is therefore unclear if positive aspects of the technique can mitigate these limitations.

This study reported on the acceptability of UVBF versus standard treatment as part of a pilot feasibility trial (Cleland et al., 2022). Results met our objectives: in both intervention arms, more than 75% of families rated randomization as acceptable in a questionnaire. The questionnaire also showed that more than 75% of families rated UVBF as acceptable and that articulation intervention (standard care) was also acceptable, with the caveat that around half of participants would not wish to continue the articulation intervention after the study, often because they had other priorities or did not feel further intervention was necessary. The qualitative analysis revealed that there were more positive than negative themes regarding acceptability, particularly participants’ affective attitudes where high levels of enjoyment were expressed in both groups. This, together with the quantitative results suggests that criteria for moving to a full trial are met. Below we expand on these positive findings alongside highlighting the negative themes expressed by participants with suggestions for mitigation of these.

### Positive themes: Ethicality, affective attitude, perceived effectiveness, self‐efficacy and intervention coherence

In both intervention arms participants expressed especially positive affective attitude. While in the articulation group, this was mainly related to enjoying the games used by the SLTs for reinforcement, the UVBF group highlighted the enjoyment of seeing tongue movements on the computer screen. This perhaps emphasizes that in an articulation intervention session, the SLT's skill in applying the principles of motor learning are not necessarily obvious to a family, whereas the board games or tabletop activities they use to reinforce this may be. This contrasts with ultrasound when the child's attention is drawn to the mechanism of action of the intervention: the biofeedback of tongue movements. The novelty value of ultrasound was therefore high and the intervention coherence is clear to families, whereas it may be less so in articulation intervention. Both the questionnaire and the focus group highlighted a benefit unique to ultrasound: being able to see the tongue movements on a screen and from this gain knowledge about articulation. This is in line with Dugan et al. (2023) who found that clinicians rated this as the largest benefit of UVBF.

Both groups valued the interpersonal relationship with the SLTs. This is in contrast with Alighieri et al. (2023) who found that only families who received high‐intensity intervention (which this was not) valued the patient–therapist relationship. This is perhaps because in this study the intervention was carried out by SLTs who had a previous working relationship with almost all the families. For both groups, the study was worthwhile (articulation group) and they would recommend it to others (ultrasound group), fitting with participants’ ethicality (Sekhon et al., 2017). This is encouraging from the point of view of a future clinical trial. While this study did not measure effectiveness, again both groups mentioned perceived improvements (perceived effectiveness) in speech production, though in the case of the ultrasound group, some highlighted that these changes were minimal. This is not surprising given the fact that the intervention duration was short and our original protocol did not predict widespread generalization (Cleland et al., 2022). Moreover, in both groups, a need for further sessions and a willingness to attend them was mentioned. Participants clearly articulated that there were improvements in speech. This aligns with previous studies which suggest that speech intelligibility is an important outcome for these families (Alighieri et al., 2021; Alighieri, Peersman et al., 2020; Sell et al., 2023). It was also interesting to note that the ultrasound group valued an improved understanding of how speech sounds are formed gained from ultrasound imaging, which is a potential advantage of this technique. Improving parents’ knowledge is an important and powerful tool in speech intervention (Sell et al., 2023).

While most of the intervention was delivered by the SLTs, in terms of self‐efficacy the parents in the articulation therapy group expressed that they were able to practice outwith sessions, though this is burdensome and the parents of the children in the ultrasound group again reiterated improved knowledge of speech production. This aligns with Sell et al. (2023) who through training parents to deliver intervention at home reported that parents grew in knowledge, skill and insight.

### Negative themes: Burden, opportunity costs, affective attitude and perceived effectiveness

The key burden for both groups was travel. This aligns with the study by Alighieri et al. (2023). Although the hospital (and also the university) is in a city centre with good transport links, the craniofacial service serves a large geographical area, much of which is rural and one parent in the ultrasound group commented that it was a 4‐hour round trip for her family. This is clearly a negative effect of centralizing services and participants suggested that having more local locations or undertaking the intervention in schools could be a good solution. Interestingly, while in previous studies telehealth has been suggested as a solution (Alighieri et al., 2023; Sell et al., 2023; Southby et al., 2022), this was not suggested by participants in our study, with one parent in the articulation group suggesting her child preferred a face‐to‐face appointment. It should also be noted that the availability of ultrasound equipment is a potential barrier to delivering this type of intervention in a greater number of locations (Dugan et al., 2023) and that articulation intervention would be much more feasible over telehealth than UVBF which would require each participant to have their own device. A further burden was the childcare of other siblings, which is particularly difficult for single‐parent families to manage. The articulation group also commented on the burden of homework/practice which can be difficult to fit into home life. This echoed the study by Sell et al. (2023) which reported that parents found it difficult to find the time to carry out home‐based activities.

Although above we highlight the positive affective attitude of participants, there were some comments in the articulation group that the therapy was boring and repetitive. This intervention is drill based and Alighieri et al. (2021) also found that their intervention lacked variety. It is also worth noting that in our study dosage, that is number of repetitions of a target speech sound or word, was standardized across interventions, so both interventions are drill‐based. The novelty of the ultrasound image could therefore be an advantage, particularly for children who have had previous articulation therapy.

Taking part in an intervention study also comes with an opportunity cost. Alighieri et al. (2023) found that children in their study had to miss out on hobbies and birthday parties. In our study, we offered as much flexibility in appointments as possible, with some sessions rescheduled at participants’ request to allow, for example, school trips. However, many children came to appointments during school time and this was an issue for one parent in the articulation group. Nevertheless, this is countered with positive comments about the importance of speech.

## LIMITATIONS

A key limitation of this study is the sample size: both the overall group and the limited number of participants (due to recruitment issues which will be discussed elsewhere), particularly children, who came forward for the online focus groups. This limitation is potentially exacerbated by the wide age range of children included. Nevertheless, the concordance between the questionnaires and the focus groups suggests that our overall findings are representative of the families who took part. In this study we chose to invite only children over the age of 12 to give their views, this resulted in very limited recruitment because most of the participants in this study were under this age (this was not known at the outset of the study). A solution to this would be to use different methods to collect the views of younger children (Owen et al., 2004). Another key omission in this study was the views of people who chose not to participate at all. The theoretical framework of acceptability (Sekhon et al., 2017) highlights the perceived *anticipated* acceptability of interventions, for example, it is possible that people who chose not to take part in the study did so because they perceived the burden too high, or they did not believe the interventions would be effective. Future trial planning could incorporate the views of these people to find further solutions to increase recruitment.

We were also unable to report whether children had had extensive speech therapy in the past. It is likely that many of the children had received articulation intervention in the past and this therefore gave ultrasound a benefit as a novel intervention, indeed one parent commented that articulation intervention was ‘just the usual’. To allow us to fully understand the impact prior intervention choices might have had on families’ views of both interventions it would be useful in future studies to access speech and language therapy records and report prior experiences.

Finally, in this report of the study and in our original protocol we did not seek the views of the clinicians carrying out the intervention. This is important because delivering novel interventions in a clinical trial carries a burden. We will report in a future study the views of the SLTs who carried out these interventions alongside the perceived anticipated acceptability of a trial of UVBF versus articulation therapy in a wider group of SLTs.

## CONCLUSIONS

In conclusion, both UVBF and articulation therapy are acceptable and indeed enjoyable for families of children with CP±L. Clinicians can be assured when offering these interventions that families will have a positive affective attitude towards them and that both interventions are well tolerated. Randomization to either UVBF or articulation therapy is acceptable to families and the burden of the additional research outcome measures required for a clinical trial are manageable. Both interventions carry a burden, particularly travelling to the hospital location, but this is not unique to these interventions. Potential solutions include carrying out the interventions in schools, having longer or more sessions to ensure the therapy is as effective as possible, or carrying out the interventions in more locations. The results of this study support the need to carry out a full‐scale randomized control trial to determine the effectiveness of UVBF compared with articulation therapy for children with CP±L.

## CONFLICT OF INTEREST STATEMENT

The authors report no conflicts of interest.

## PATIENT CONSENT STATEMENT

All participants gave informed consent.

## Data Availability

The data that support the findings of this study are available from the corresponding author upon request. The data are not publicly available due to privacy or ethical restrictions.

## ENDNOTES

1 Two participants were siblings and the parents completed one questionnaire applying for both.

2 When checking the data, the treating clinicians stated that stickers were not used during the intervention. These were, however, used for reinforcement at the university during the pre‐ and post‐assessments.
